# Supplementary material for: A UMLS-based spell checker for natural language processing in vaccine safety
Source: BMC Med Inform Decis Mak. 2007 Feb 12;7:3. doi: 10.1186/1472-6947-7-3 (PMC1805499; doi:10.1186/1472-6947-7-3)
Supplement: Additional file 13 — RAP application directory. Sets up directory for RAP (RDF application for PHP) source code [file 1472-6947-7-3-S13.gz › rap/doc/phpdoc/__filesource/fsource_dataset__datasetNamedGraphDb.php.html]

xml version="1.0" encoding="iso-8859-1"?


File Source for NamedGraphDb.php


# Source for file NamedGraphDb.php

Documentation is available at NamedGraphDb.php

```
1. <?PHP
2. // ----------------------------------------------------------------------------------
3. // Class: NamedGraphDb
4. // ----------------------------------------------------------------------------------


8. /**
9. * Persistent NamedGraph implementation that extends a {@link DbModel}.
10. * The graphName is not stored in the database. As soon as the namedGraph is added
11. * to a RDF dataset the graphName is saved.
12. *
13. * <BR><BR>History:<UL>
14. * <LI>05-30-2005                : First version of this class.</LI>
15. *
16. * @version  V0.9
17. * @author Daniel Westphal (http://www.d-westphal.de)
18. *
19. * @package     dataset
20. * @access    public
21. ***/
22. class NamedGraphDb extends DbModel
23. {

25. /**
26. * Name of the NamedGraphDb
27. *
28. * @var        string
29. * @access    private
30. */
31. var $graphName;

33. /**
34. * Constructor
35. * Do not call this directly.
36. * Use the method getModel,getNewModel or putModel of the Class NamedGraphDb instead.
37. *
38. * @param   ADODBConnection
39. * @param   string
40. * @param   string
41. * @param   string
42. * @access    public
43. */
44. function NamedGraphDb(&$dbConnection, $modelURI, $modelID,$graphName, $baseURI=NULL)
45. {
46. $this->dbConn =& $dbConnection;
47. $this->modelURI = $modelURI;
48. $this->modelID = $modelID;
49. $this->baseURI = $this->_checkBaseURI($baseURI);
50. $this->graphName = $graphName;
51. }

53. /**
54. * Sets the graph name.
55. *
56. * @param string
57. * @access    public
58. */
59. function setGraphName($graphName)
60. {
61. $this->graphName=$graphName;
62. }

64. /**
65. * Return the graph name.
66. *
67. * @return string
68. * @access    public
69. */
70. function getGraphName()
71. {
72. return $this->graphName;
73. }
74. }
75. ?>
```

Documentation generated on Thu, 7 Jul 2005 13:42:15 +0200 by phpDocumentor 1.3.0RC3
